# Growth-coupled selection of synthetic modules to accelerate cell factory development

**DOI:** 10.1038/s41467-021-25665-6

**Published:** 2021-09-06

**Authors:** Enrico Orsi, Nico J. Claassens, Pablo I. Nikel, Steffen N. Lindner

**Affiliations:** 1 Max Planck Institute of Molecular Plant Physiology, Potsdam-Golm, Germany; 2 Laboratory of Microbiology, Wageningen University, Wageningen, The Netherlands; 3 The Novo Nordisk Foundation Center for Biosustainability, Technical University of Denmark, Kongens Lyngby, Denmark

**Keywords:** Metabolic engineering, Metabolic engineering, Synthetic biology

## Abstract

Synthetic biology has brought about a conceptual shift in our ability to redesign microbial metabolic networks. Combining metabolic pathway-modularization with growth-coupled selection schemes is a powerful tool that enables deep rewiring of the cell factories’ biochemistry for rational bioproduction.

The field of metabolic engineering has entered the next level of maturity, shifting from looking at metabolism as a rigid structure to a rather flexible and versatile network. Recently, several groundbreaking studies focused on central assimilation pathways have demonstrated an unprecedented level of control over core metabolic networks. Examples of this sort aimed at establishing alternative assimilation pathways in microbial workhorses, e.g., implementing the autocatalytic Calvin–Benson–Bassham and ribulose monophosphate cycles in *Escherichia coli* and *Pichia pastoris*^1–3^. Their realization required the steady-state maintenance of appropriate concentrations of intermediate metabolites to allow for the activity of these non-native metabolic routes^4^. Moreover, an equally important rewiring of central metabolism in *E. coli* led to a non-oxidative glycolysis^5^. The resulting strain could catabolize one glucose equivalent to three acetyl-CoA molecules, instead of the two obtained via the canonical glycolysis. Non-traditional hosts can now also be engineered by harnessing the enabling tools of synthetic biology, as illustrated by the implementation of a linear glycolysis in the soil bacterium *Pseudomonas putida*, which fully replaces the native Entner–Doudoroff catabolism^6^. The combination of rational engineering and adaptive laboratory evolution (ALE) has been crucial to achieve these deep metabolic reconfigurations. The success of such studies shows a surprising degree of metabolic plasticity, even in core routes, which challenges the established textbook knowledge describing central metabolism as a static network.

The degree of control we can now exert over metabolic networks can be exploited for designing next-generation cell factories. The societal impact of industrial biotechnology depends significantly on our ability to “teach” microbial hosts how to efficiently convert a substrate into a product of interest. TRY values, i.e., titers (mmol_product_ · L^−1^ or *g*_product_ · L^−1^), rates (*g*_product_ · *g*_CDW_^−1^ · h^−1^ or *g*_product_ · L^−1^ · h^−1^), and yields (*g*_product_ · *g*_substrate_^−1^ or *g*_product_ · *g*_CDW_^−1^), are the benchmarks of product formation efficiency^7^. One way to increase TRYs is engineering microbial metabolism to funnel carbon flow into the target bioproduction pathways. Traditional metabolic engineering strategies for improving production pathway capacities include overexpression of required pathway enzymes for a desired product and knockout of enzymes competing for metabolites with the production pathway. These genetic engineering interventions are typically followed up by screening strain performances; such traditional approaches have been extensively reviewed elsewhere^8^. In this study, we discuss the rationale behind (re-)emerging strategies for improving cell factories and propose exploiting metabolic versatility to advance the state-of-the-art.

## Synthetic metabolism: modularity meets growth-coupled design

We advocate that metabolic fluxes can be rewired by employing rationally designed selection strains as an alternative to traditional metabolic engineering approaches. In literature, such an approach is usually described as “growth-coupled selection”^9^ or “growth-based selection”^10^. Here, metabolism is strategically interrupted by gene deletions and growth under restrictive conditions is exclusively rescued upon flux through the target enzyme or pathway^11,12^. When maintaining such a selective pressure, selection strains can also evolve, thereby increasing flux capacity through the enzyme(s) of interest. In other words, combination of rational design, growth-coupled selection, and ALE will pave the way for screening and improving enzyme and pathway variants, which can be transformed into a microbial production platform. The coupling of metabolite biosynthesis to growth has been harnessed as a successful bioproduction strategy already for very long, especially in anaerobic fermentations. In the last two decades, design of knockout strains to couple production of new molecules to growth has been extensively explored, both by modeling and experimental approaches^13,14^. Yet, growth-coupled selection of pathway modules is still a relatively underrated approach for metabolic engineering.

**Fig. 1:**
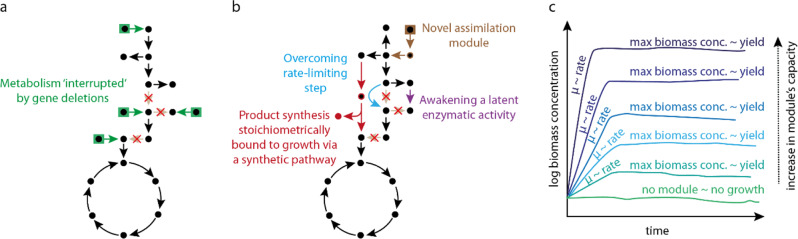
Schematic representation of the metabolic network of growth-coupled selection strains and their role as proxy for determining rate and yields. Black circles represent essential intermediates that act as biomass precursors. **a** Selection strain, where gene deletions “slice” metabolism and microbial growth becomes dependent on the supplementation of external substrates (green boxes). **b** Possible use of selection strains for testing different types of metabolic modules. This representation condenses all module types that can (independently) rescue growth. Each arrow color represents a particular type of module that can be screened via this approach: (i) novel assimilation route (brown); (ii) metabolic bypass for avoiding a rate-limiting step (light blue); (iii) exploration of latent promiscuous enzymatic activities (purple); and (iv) (synthetic) pathway that also produces a compound of interest (red). **c** Module activities will result in growth of the selection strain; monitoring biomass formation provides information related to rates and yields.

Modularity is the enabling concept for the design and harnessing of selection strains. Following the synthetic biology approach for pathway engineering, we can divide metabolic routes into functional modules consisting of at least one enzymatic activity^15,16^. Then, to assess these modules in vivo, we can test and optimize them into dedicated microbial selection strains. When no module is present, these strains depend on supplementation of additional nutrients for synthesizing one or more biomass precursors^17^ (Fig. 1a). Modular selection strains are designed as such that, when removing external nutrient additions, synthesis of biomass building blocks relies solely on the activity of the tested module^18^ (Fig. 1b). The advantage of this approach is that the module’s functionality is directly coupled to biomass formation^11^. Therefore, growth monitoring can be used to estimate the relative performance of different module variants in terms of the following: (i) rate, which can be approximated by assessing the growth rate of the selection strains, and (ii) yield, for which the highest biomass concentration of the selection strain can serve as proxy (Fig. 1c).

The stringency on the strain’s metabolic selection can be increased by introducing additional gene deletions or manipulating the incubation conditions, e.g., by changing or removing carbon substrates that feed different nodes in the biochemical network. This influences the concentration of biomass precursors, i.e., affecting maximal yield, and hence the flux the module needs to support^11,17^. As explained above, ALE can further improve the module’s functionality. A successful example of the step-by-step use of multiple selection strains with increasing stringency was the establishment of the complete formate assimilatory reductive glycine pathway in two separate microbial chassis^19,20^.

In summary, by carefully “slicing” the metabolism into functional blocks to construct dedicated selection strains, multiple platforms can be generated for testing and tinkering with a variety of metabolic modules. Enzyme or pathway libraries can be assessed within this context, replenishing carbon in the essential metabolic nodes excluded by the gene deletions. Moreover, with this strategy “latent” enzymatic activities present in native host can be awakened^21–24^, as well as new-to-nature ones could in principle be discovered^25^ (Fig. 1b). Using growth as an in vivo readout, optical density becomes a straightforward means to functionally assess module candidates (Fig. 1c). In the next section, we discuss how this fast and simple monitoring tool can be used as an analytical step for high-throughput enzyme or pathway screenings and engineering.

## Accelerating strain optimization using growth-coupled design

While entering its third decade of life as an applied discipline^26^, synthetic biology claims an evident contribution to biomanufacturing^27,28^. The current SynBio paradigm for rational strain engineering is defined by the “design-build-test-learn” (DBTL) cycle^7^. By iterating DBTL cycles, microbial strains are improved in their TRY values. Product formation must be measured, and many research endeavors have significantly improved the accessibility and the rapidity of high-throughput, -*omics* pipelines for metabolites determination^29,30^. However, the type and number of samples to prepare, analyze, and interpret can render the analytic step a bottleneck in some DBTL cycles^31,32^. Only in very few cases product formation is measurable via readily available, cost-effective readouts, such as, e.g., colorimetry^33^.

**Fig. 2:**
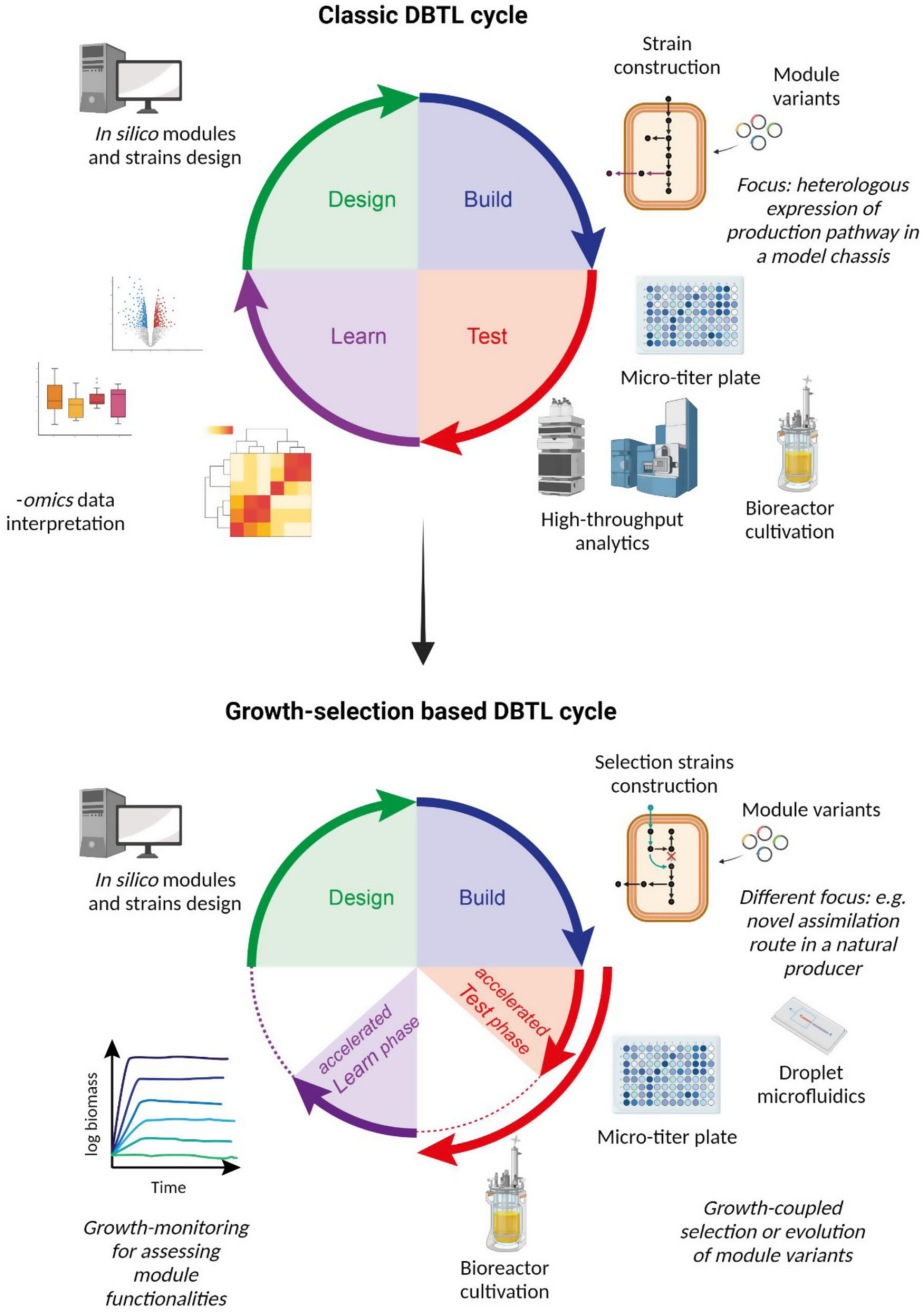
The classical “design-build-test-learn” (DBTL) pipeline (top) compared to the “growth selection-based” DBTL cycle proposed in this study (bottom). The “design” and “build” phases are supported by the same technologies in both cycles. Nevertheless, the novel pipeline changes in the “test” and “learn” phases, which become faster in their execution due to the lack of -*omics* analyses (shorter red and purple arrows). In case of necessity, the new cycle can host adaptive laboratory evolution within its “test” phase (longer red arrow). Created with BioRender.com.

If bioproduction can be coupled to the growth of a selection strain (Fig. 1b), any “simple” measurement of biomass formation will be a readout of product synthesis. More generally, any selection strain investigated via growth-coupled schemes could represent a cost-competitive platform to rapidly test a module’s performances. For such a pipeline, the DBTL paradigm can be simplified accordingly (Fig. 2). The “design” phase may now also consist of in silico planning of the gene deletions to implement in one or more selection strains. Moreover, at this stage, one can choose whether test a library of module variants or mutate the DNA target sequence of the inserted pathway genes e.g., via error-prone PCR, multiplex automated genome engineering (MAGE), or clustered regularly interspaced short palindromic repeats (CRISPR)-Cas technologies^34^. In the “build” phase, the selection strain will be generated and the module(s) of interest (and variants) will be plugged into the strain. In the “test” phase, the different strains will be cultivated under one or more selective conditions, and biomass formation will be used as a proxy for in vivo quantification of modules’ rates and yields (Fig. 1c). Then, in the “learn” phase, growth data acquisition and interpretation will determine whether the results obtained are satisfactory, i.e., whether the modules tested are performing well enough or whether a new cycle must be initiated. If a library of variants is tested, gene sequencing at the end of the cultivation will help to identify the best-performing module variant. In case the tested module underperformed, the designer could decide to modify the module’s architecture or the stringency of the selection schemes^17^.

A key advantage of this novel pipeline is that, in case of need, the “test” phase can be adapted into a short- or long-term ALE to increase the module’s capacity. This can be performed with the very same selection strains. Once a DBTL cycle ends with evolution, the “learn” phase will include whole genome sequencing of the improved strain. This will allow to understand the pattern of mutations, which can be retro-engineered individually or in combination in a new DBTL cycle to assess their role in improving the growth phenotype.

We argue that this adapted “growth selection-based” DBTL concept (Fig. 2) improves the state-of-the-art by avoiding the current “test” phase bottleneck of some high-throughput strain engineering endeavors (i.e., -*omics* analyses), while still providing straightforward and useful data about the performance of biological parts. Moreover, it is a standard process that can be adapted to different needs, such as testing novel assimilation routes, alternatives for rate-limiting enzymes in the central metabolism, or synthetic production pathways (Fig. 1). One example of application of this DBTL pipeline is illustrated by an *E. coli* NADPH-“auxotroph” strain, which was created for in vivo testing of NADPH regenerating reactions^35^. Such a platform served for identifying mutated formate dehydrogenases with NADP specificity^36^. If a full production pathway is strictly connected to growth, its growth-coupled selection can also be a useful analytic tool for determining the best-performing pathway variants and enhancing productivity. Yet, the proposed selection-based DBTL approach can also be employed in case the full production pathway cannot be coupled to growth. In fact, important key steps upstream of the production pathway itself (e.g., rate-limiting steps or alternative substrate assimilation pathways) could be optimized by using this pipeline. One exemplary study that used such growth-coupled approach for upstream pathway optimization tackled the production of N-hexanol in *E. coli*^37^. To optimize the upper part of the pathway towards C6-precursors formation, the authors coupled NAD^+^ regeneration under anaerobic conditions to the formation of hexaonic acid. This served as a base to implement the whole pathway towards *n*-hexanol. In summary, use of growth-coupled selection could optimize production by performing early DBTL cycles and later continue with classical product screening-based DBTL cycles. This approach demonstrated to be beneficial, as the number of variants to be screened via the later classic DBTL could be limited.

## An alternative approach for improving cell factories

Biofoundries are high-throughput infrastructures used for analysis and optimization of biological systems via DBTL cycles^32,38^. In a biofoundry, rapid prototyping and optimization generally focuses on the production pathway of a cell factory. However, due to their value as high-throughput infrastructure, they are finding alternative applications to the original DBTL pipeline for biomanufacturing^39^. Because of its technical simplicity, growth-coupled selection is an interesting setup for streamlining the DBTL method in biofoundries. As an intellectual exercise, here we describe how the biofoundry pipeline could be adapted based on this very approach (Fig. 2). For this purpose, biofoundries need to be properly equipped to characterize growth of many strains in parallel (i.e., using micro-titer plates or droplet microfluidics^40^) or allow for the selection of the fastest growers in direct competition cultures (e.g., in controlled bioreactors). Such a pipeline can be employed for screening from single enzymes up to entire pathways. We argue that one can follow this method for the assessment of candidate modules needed for substrate assimilation, as well as testing alternatives for debottlenecking rate-limiting enzymatic steps within the central carbon metabolism. A complementary utilization of such infrastructures could include selecting microbial platforms that are naturally endowed with production traits of interest (e.g., synthesis of an added value compound at high titer) and then engineering alternative assimilation routes for abundant, renewable substrates (e.g., CO_2_ and H_2_, formate, or methanol). Alternative uses of these infrastructures could include testing libraries of enzyme variants that are rate limiting in central carbon metabolism or introducing synthetic pathways that better connect key metabolic nodes important for microbial fitness. In conclusion, the use of growth-coupled selection in combination with pathway modularization and evolution represents an accessible, highly valuable opportunity for expanding the boundaries of industrial biotechnology by creating superior cell factories for novel bioprocesses.
